# Circulation and overwintering of Usutu virus lineages in north-eastern Spain: A one health perspective (2021–2025)

**DOI:** 10.1016/j.onehlt.2026.101400

**Published:** 2026-04-01

**Authors:** Arjola Leka, Jaume Gardela, Elena Obón, Maria Pifarré, Miguel Julián Martínez, Jéssica Navero-Castillejos, Ramon Casals, Alba Solé, Núria Busquets

**Affiliations:** a IRTA. Programa de Sanitat Animal, Centre de Recerca en Sanitat Animal (CReSA), Campus de la Universitat Autònoma de Barcelona (UAB), Bellaterra, Catalonia, Spain; b Unitat mixta d'Investigació IRTA-UAB en Sanitat Animal, Centre de Recerca en Sanitat Animal (CReSA), Campus de la Universitat Autònoma de Barcelona (UAB), Bellaterra, Catalonia, Spain; c Centre de Fauna de Torreferrussa, Àrea de Gestió Ambiental Servei de Fauna i Flora, Forestal Catalana, Santa Perpètua de Mogoda, 08130, Spain; d Centre de Fauna dels Aiguamolls de l'Empordà, Àrea de Gestió Ambiental Servei de Fauna i Flora, Forestal Catalana, Castelló d'Empúries, 17486, Spain; e Department of Clinical Microbiology, Hospital Clínic, Barcelona, Spain; f Centro de Investigación Biomédica en Red de Enfermedades Infecciosas (CIBERINFEC), Instituto de Salud Carlos III, Madrid, Spain; g Barcelona Institute for Global Health (ISGlobal), Hospital Clinic de Barcelona, Universitat de Barcelona, Barcelona, Spain; h Secretaria de Salut Pública, Departament de Salut, Generalitat de Catalunya, Barcelona, Spain; i Departament d'Agricultura, Ramaderia, Pesca i Alimentació Generalitat de Catalunya, Servei de Sanitat Animal, Barcelona 08007, Spain

**Keywords:** Arbovirus, Usutu virus, Vector-born disease, Zoonosis, Animal health, Human health, Surveillance

## Abstract

Usutu virus (USUV) is a mosquito-borne flavivirus that has expanded widely across Europe. While it has been traditionally considered an animal pathogen, reports of human infections and the detection of USUV in blood donors in Europe (including Spain) have increased the public health awareness of this emerging pathogen. In the last decade, serological evidence of USUV circulation in animals have been reported in Northeastern Spain (Catalonia) where human infections have also been recently detected; however, molecular confirmation of viral circulation in wild birds was still lacking. Our main goal was to investigate USUV circulation in Catalonia (2021–2025) using One Health approach through the West Nile virus surveillance program. Once USUV was detected, we aimed to assess USUV overwintering, genetic diversity and pathology in positive wild birds. From 369 bird samples collected, six blackbirds from the Barcelona province resulted USUV-positive in 2022, 2024, and 2025, showing signs of systemic infection. Additionally, USUV was found in a *Culex pipiens* mosquito pool, and sentinel and backyard birds showed serological evidence of flavivirus exposure in Girona province. Phylogenetic analyses revealed USUV overwintering, evidence of human spillover and the co-circulation of two distinct lineages, Africa 3 and Europe 2. Notably, Europe 2 was detected for the first time in Spain. These findings prove active enzootic USUV circulation in Northeastern Spain and highlight the need for One Health surveillance to support early USUV detection to reduce the risk of USUV transmission from enzootic cycle.

## Highlights

• Integrated One Health monitoring reveals USUV enzootic transmission North-Eastern Spain.
• Phylogenetic analysis confirms human spillover, rather that a new introduction.
• Co-circulation and overwintering of USUV Africa 3 and Europe 2 lineages.
• First report of Europe 2 lineage in Spain and its westward expansion in Europe.

## 1 Introduction

Usutu virus (USUV) (*Orthoflavivirus usutuense*) is an arbovirus of the family *Flaviviridae* [1] and a member of the Japanese encephalitis virus antigenic complex, along with several other human and animal pathogens such as West Nile virus (WNV). USUV is maintained in nature through an enzootic transmission cycle involving mainly *Culex* mosquito vectors and wild birds as amplifiers [2]. USUV is highly pathogenic to several birds, particularly within the orders Passeriformes and Strigiformes [3], [4]. In humans, USUV infection is generally asymptomatic or mild, but sporadic cases of severe neurological symptoms or even death have occurred [5].

Since its first European detection in Austria in 2001 [6], USUV has been detected across Europe [7]. Phylogenetic analyses have shown that USUV likely spread from Africa to Europe through avian migration [8] and has been classified into eight distinct lineages: three African (Africa 1–3) and five European (Europe 1–5). All lineages, except Africa 1, have been reported in Europe [9]. The widespread circulation of USUV across Europe suggests the persistence of transmission cycles in affected areas, potentially sustained by the overwintering of infected mosquitoes or repeated reintroductions of the virus, but also through persistent infections in avian hosts [10].

Since the first USUV detection in Catalonia in 2006 [11] radically reported in mosquitoes and wild birds in different regions of Spain [12]. Recently, human infections have been identified, including three blood donors (one blood donor from Majorca and two from Catalonia) [13]. In Catalonia, serological studies conducted over the last decade indicated USUV circulation [14]. However, no molecular evidence of USUV circulation had been reported in the region since its initial detection in 2006 [11] until the recent identification of USUV in blood donors [13]. The present study investigates the circulation of USUV in Catalonia between 2021 and 2025, capitalizing on existing WNV-surveillance capacities. The main goal of this work is to increase our understanding of USUV spillover events in humans. This is achieved by expanding our knowledge on USUV epidemiology, overwintering, and pathology in local bird populations.

## 2 Materials and methods

### 2.1 Samples selection and collection

Samples were obtained through the Catalan WNV Surveillance Program [15], within the national surveillance framework [16]. This program is implemented under a One Health approach through coordinated collaboration between the Animal Health Prevention Service (Department of Agriculture, Livestock, Fisheries and Food, Government of Catalonia), responsible for animal surveillance, and the Environmental Health Service (Department of Health), responsible for entomological surveillance. Collection targeted wild, sentinel, backyard birds, and mosquitoes throughout the mosquito activity season. Ethical approval was not required, as no trapping and sampling of wild birds was performed in this study. All samples from wild birds were obtained from birds found dead or showing clinical signs that were submitted at the Wildlife Recovery Centres (WRC) in the frame of routine surveillance for public health authorities. All procedures followed approved guidelines, regulations and the principles of the Declaration of Helsinki.

Brain samples were extracted under biosafety level 3 (BSL-3) conditions at IRTA-CReSA facilities, initially analyzed for WNV. Based on previous European reports of USUV infection in wild birds, 369 birds from 31 species and 11 orders, collected between 2021 and 2025 (Fig. S1A), were selected for USUV testing (Fig. S1B).

**Fig. 1:**
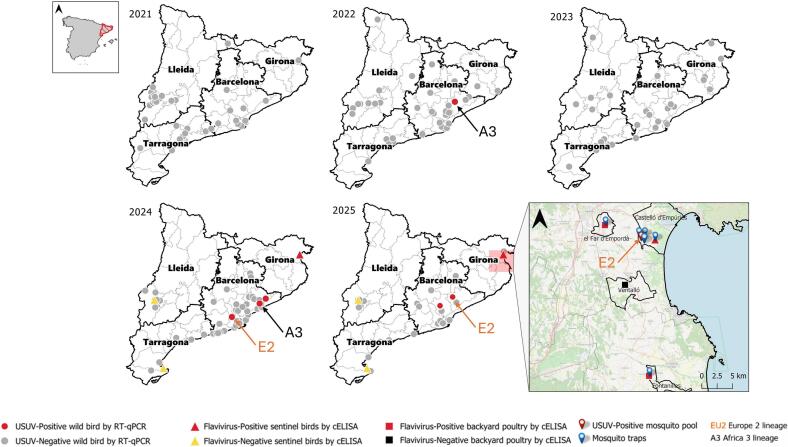
Spatiotemporal distribution of USUV-positive and USUV-negative wild bird samples (2021–2025), together with flavivirus-positive and flavivirus-negative sentinel birds (2024–2025), flavivirus-positive and flavivirus-negative backyard birds (2025), mosquito traps location (2024–2025), as well as location of USUV-positive mosquito pool in 2025. A3 indicates the Africa 3 lineage, while E2 indicates the Europe 2 lineage.

Sentinel birds were included in 2024 in three WRC (*Centre de Fauna de Vallcalent* (VC), *Estació Biològica de Canal Vell* (CV) and *Centre de Fauna dels Aigüamolls de l'Empordà* (AE)), involving 23 sentinel birds (*Gallus gallus*) distributed across three provinces: Girona (34 serum samples from five sentinel chickens were collected from May to December), Lleida (24 serum samples from 10 sentinels were collected from June to October), and Tarragona (64 serum samples from eight sentinels were collected from April to November) (Fig. 1).

In 2025, 10 sentinel chickens (four retained from 2024 and six newly introduced ones that replace the old ones successively after spring) and 70 backyard poultry (60 *Anas* sp. and 10 *Gallus gallus* broilers), which were sampled at the slaughterhouse, were collected in areas with previous flavivirus circulation (Fig. 1).

Mosquito sampling was performed in areas with previous flavivirus circulation in sentinel chickens and backyard poultry, as well as in locations where traps were routinely placed within WNV-surveillance program near to wetlands by the *Servei de Control de Mosquits de la Badia de Roses i el Baix Ter* (Fig. 1). Mosquito traps operated 18–24 h every 14 days. Collected mosquitoes were morphologically identified to specie level. Females were pooled by species, collection site and date (up to 25 individuals per pool).

### 2.2 Molecular and immunohistochemical detection of USUV

Brain samples were homogenized by using polypropylene pestles. The same procedure was applied to different tissues collected from one blackbird collected in 2025, to assess viral distribution. Mosquito pools were homogenized by using a bead mill (TissueLyser II, Qiagen, Denmark) at 30 Hz for 1 min. Viral RNA was extracted using NucleoSpin® RNA Virus (Macherey-Nagel, Düren, Germany), following the manufacturer's instructions. USUV RNA was detected by a previously designed USUV-specific real-time RT-qPCR [17]. Reactions were carried out using the AgPath-ID™ OneStep RT-PCR Kit (Applied Biosystems, Foster City, CA, USA) on a 7500 Fast Real-Time PCR System (Applied Biosystems, Foster City, CA, USA).

Immunohistochemical detection of USUV antigen in bird tissues (brain, liver, heart, lungs, spleen, kidney, intestine, stomach) was performed as previously described [18], using the flavivirus anti-NS1 antibody [D2D6B7] (ab214337, Abcam).

### 2.3 Serological detection of flavivirus and USUV circulation

Serological analyses were performed using a commercial competitive enzyme-linked immunosorbent assay (cELISA; INgezim West Nile COMPAC R.10.WNV.K3), following the manufacturer's instructions. Positive or doubtful samples were confirmed by plaque serum neutralization test (SNT) at the Central Veterinary Laboratory (Algete, Spain), accordingly to the WOAH Terrestrial Manual [19]. The SNT was used to identify specific antibodies against WNV, USUV and Bagaza virus (BAGV).

### 2.4 USUV isolation, sequencing and phylogenetic analyses

USUV isolation from positive bird samples, total RNA extraction, library preparation and whole genome sequencing were performed as previously described [20]. Mosquito sequencing followed Siljic, et al., [21] and Sanger sequencing was performed. The GenBank accession numbers are the following: PX453694, PX453695, PX453696, PX883765 and PX955310.

Whole-genome sequences of USUV were retrieved from the NCBI GenBank database (doi:https://doi.org/10.1093/nar/gkp1024), including 67 sequences selected to provide broad representation of USUV diversity. Multiple sequence alignment was performed using MAFFT version 7 [22], [23]. Phylogenetic analysis was conducted using the Maximum Likelihood method and Tamura-Nei (1993) model [24] in MEGA v12 [25], with 1000 bootstrap replicates.

BLAST2seq was used to assess the similarity of newly obtained sequences from 2025.

## 3 Results

### 3.1 USUV molecular detection in wild birds

Among 369 bird samples collected in Catalonia between 2021 and 2025, USUV RNA was detected by RT-qPCR in brain sample of six resident blackbirds (1.63%) (Table 1), collected in different municipalities of Barcelona province (Fig. 1). These detections confirmed USUV-circulation in Catalonia during 2022, 2024, and 2025. No USUV-positive samples were identified in 2021 or 2023. All USUV detection occurred between early August and late September and Ct values ranged from 17.36 to 33.21 (Table 2). In one blackbird detected in 2025 (AC2176) all tested organs and swab samples were positive. The highest viral loads were detected in feathers, lungs, spleen and eyes; moderate levels in the cerebellum, kidney, liver, small intestine and stomach; and the lowest in the spinal cord, oral and cloacal swab, and large intestine (Table 3).

**Table 1:** Results of RT-qPCR in wild bird samples. Positive samples are highlighted in bold.

Year	Order	Common name	Scientific name	Number of USUV RT- qPCR + samples	Total number of RNA samples
2021	Accipitriformes	Common buzzard	*Buteo buteo*	0	4
	Anseriformes	Mute swan	*Cygnus olor*	0	1
	Apodiformes	Common swift	*Apus apus*	0	8
	Columbiformes	Common pigeon	*Columba livia*	0	7
		Common wood pigeon	*Columba palumbus*	0	3
	Coraciiformes	European bee-eater	*Merops apiaster*	0	1
	Falconiformes	Common kestrel	*Falco tinnunculus*	0	2
	Passeriformes	European greenfinch	*Carduelis chloris*	0	1
		Hooded crow	*Corvus cornix*	0	1
		Eurasian blue tit	*Cyanistes caeruleus*	0	1
		European robin	*Erithacus rubecula*	0	1
		Barn swallow	*Hirundo rustica*	0	6
		Great tit	*Parus major*	0	2
		House sparrow	*Passer domesticus*	0	8
		Eurasian tree sparrow	*Passer montanus*	0	2
		Eurasian magpie	*Pica pica*	0	7
		Common starling	*Sturnus vulgaris*	0	10
		Eurasian blackbird	*Turdus merula*	0	1
	Piciformes	European green woodpecker	*Picus viridis*	0	1
	Strigiformes	Little owl	*Athene noctua*	0	1
		Eurasian eagle-owl	*Bubo bubo*	0	2
2022	Accipitriformes	Common buzzard	*Buteo buteo*	0	6
	Anseriformes	Mute swan	*Cygnus olor*	0	1
	Apodiformes	Common swift	*Apus apus*	0	1
	Caprimulgiformes	European nightjar	*Caprimulgus europaeus*	0	1
	Columbiformes	Common pigeon	*Columba livia*	0	14
		Common wood pigeon	*Columba palumbus*	0	4
	Falconiformes	Peregrine Falcon	*Falco peregrinus*	0	1
		Common kestrel	*Falco tinnunculus*	0	3
	Passeriformes	Carrion crow	*Corvus corone*	0	1
		European robin	*Erithacus rubecula*	0	2
		Eurasian jay	*Garrulus glandarius*	0	1
		Great tit	*Parus major*	0	1
		House sparrow	*Passer domesticus*	0	5
		Eurasian magpie	*Pica pica*	0	14
		Common starling	*Sturnus vulgaris*	0	3
		Eurasian blackcap	*Sylvia atricapilla*	0	2
		**Eurasian blackbird**	***Turdus merula***	**1**	**7**
		Song thrush	*Turdus philomelos*	0	2
	Piciformes	Great spotted woodpecker	*Dendrocopos major*	0	1
	Strigiformes	Little owl	*Athene noctua*	0	1
		Eurasian eagle-owl	*Bubo bubo*	0	1
		Barn owl	*Tyto alba*	0	1
	Suliformes	Great cormorant	*Phalacrocorax carbo*	0	1
2023	Accipitriformes	Common buzzard	*Buteo buteo*	0	4
	Charadriiformes	Yellow-legged gull	*Larus michaellis*	0	1
	Columbiformes	Common pigeon	*Columba livia*	0	10
		Common wood pigeon	*Columba palumbus*	0	1
	Falconiformes	Common kestrel	*Falco tinnunculus*	0	7
	Passeriformes	Eurasian jay	*Garrulus glandarius*	0	1
		House sparrow	*Passer domesticus*	0	15
		Eurasian magpie	*Pica pica*	0	3
		Common starling	*Sturnus vulgaris*	0	3
		Eurasian blackcap	*Sylvia atricapilla*	0	1
		Eurasian blackbird	*Turdus merula*	0	1
		Song thrush	*Turdus philomelos*	0	1
	Strigiformes	Little owl	*Athene noctua*	0	4
		Eurasian eagle-owl	*Bubo bubo*	0	1
2024	Accipitriformes	Common buzzard	*Buteo buteo*	0	1
	Coraciiformes	European bee-eater	*Merops apiaster*	0	1
	Falconiformes	Common kestrel	*Falco tinnunculus*	0	3
	Passeriformes	European greenfich	*Carduelis chloris*	0	1
		Eurasian jay	*Garrulus glandarius*	0	3
		Barn swallow	*Hirundo rustica*	0	1
		House sparrow	*Passer domesticus*	0	7
		Eurasian magpie	*Pica pica*	0	68
		**Eurasian blackbird**	***Turdus merula***	**3**	**5**
	Piciformes	European green woodpecker	*Picus viridis*	0	1
	Strigiformes	Eurasian eagle-owl	*Bubo bubo*	0	2
	Suliformes	Great cormorant	*Phalacrocorax carbo*	0	1
2025	Charadriiformes	Yellow-legged gull	*Larus michaellis*	0	6
	Coraciiformes	European bee-eater	*Merops apiaster*	0	3
	Passeriformes	Eurasian jackdaw	*Corvus monedulada*	0	1
		House sparrow	*Passer domesticus*	0	1
		Eurasian magpie	*Pica pica*	0	63
		**Eurasian blackbird**	***Turdus merula***	**2**	**4**
Total				**6**	**369**

**Table 2:** RT-qPCR Ct-values in brain samples of USUV-positive blackbirds detected in different municipalities of Barcelona (2022–2025).

Blackbird correlative code	Collection Date	Municipality	RT-qPCR Ct-values
AC1584	23/09/2022	L'Ametlla de Vallès	27.95
AC1986	05/08/2024	Vallgorguina	33.21
AC2018	12/09/2024	Corbera de Llobregat	18
AC2025	25/09/2024	Dosrius	24.65
AC2149	27/08/2025	Bigues i Riells	17.36
AC2176	05/09/2025	Terrassa	18.58

**Table 3:** RT-qPCR Ct-values in tissues and swabs from blackbird AC2176.

Sample	RT-qPCR Ct-values
Eyes	22.10
Cerebellum	23.63
Spinal cord	27.24
Heart	23.95
Lung	20.64
Liver	24.52
Spleen	20.09
Kidney	23.76
Stomach	23.79
Small intestine	23.85
Large intestine	37.81
Cloacal swab	29.68
Oral swab	28.03
Feather	19.90

### 3.2 Clinical and pathological findings in USUV-positive birds

The six USUV-positive blackbirds were male individuals, submitted either due to the presence of clinical signs or found dead. Neurological signs were the predominant clinical presentation in birds admitted alive, including abnormal behavior, tremors, head tilt and impaired balance (Video S1). Poor body condition or malnutrition was consistently observed at necropsy. Frequent macroscopic findings included hepatomegaly and splenomegaly, while respiratory and central nervous system lesions were observed in individual cases. Findings are presented in Table 4.

**Table 4:** Clinical signs and necropsy findings of USUV positive blackbirds.

Blackbird correlative code	Entry date	Age	Sex	Clinical signs at admission	Outcome	Pathological findings
AC1584	21/09/2022	Adult	Male	• Neurological signs (head tilt, tremors)	Died overnight	• Malnutrition (79 g) • Poor body condition (muscle score 1/3, fat score 0/8) • Mild caudal pulmonary haemorrhage • Splenomegaly with brownish discoloration • Pale kidneys
AC1986	05/08/2024	Adult	Male	• Found dead	Necropsy only	• Malnutrition • No other macroscopic lesions
AC2018	11/09/2024	Subadult	Male	• Dead on arrival	Necropsy only	• Malnutrition • No gross lesions
AC2025	25/09/2024	Adult	Male	• Neurological signs (abnormal behavior, repetitive head movements)	Euthanized	• Malnutrition (53 g) • Lesion in the left lung (not further characterized)
AC2149	27/08/2025	Juvenile	Male	• Enable to fly • Severe neurological signs (absent mental status, marked cranial tremors)	Euthanized	• Malnutrition (fat and pectoral atrophy) • Hepatomegaly • Splenomegaly • Pale areas within the encephalon • Caudal right thoracic airsasculitis with yellow fibrinous adhesion to the liver
AC2176	05/09/2025	Juvenile	Male	• Neurological signs • Severe weakness • Unable to maintain upright pοsture, presenting lateral recumbency and loss of balance	Euthanized	• Malnutrition (66 g) • Hepatomegaly • Splenomegaly

### 3.3 USUV-antigen distribution in bird tissues

**Fig. 2:**
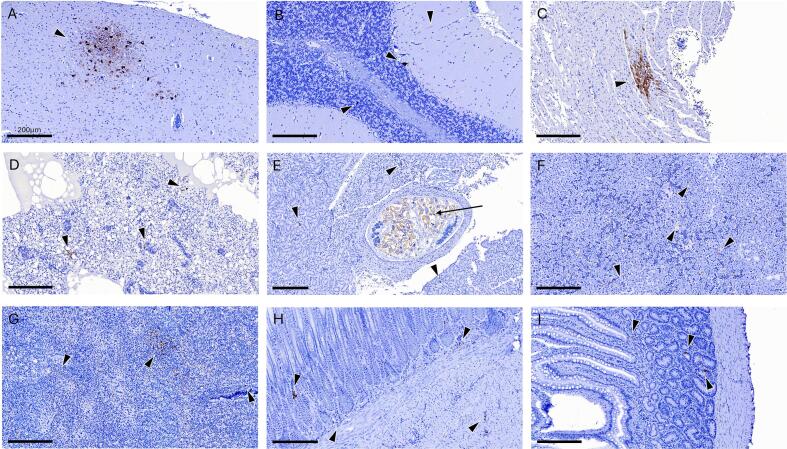
Immunohistochemical detection of Usutu virus antigens in a blackbird AC2176 (*Turdus merula*). Staining in antigen-positive cells from the (A) brain, (B) cerebellum, (C) heart, (D) lung, (E) kidney, (F) liver, (G) spleen, (H) stomach, and (I) small intestine. Arrowheads denote antigen detection, while arrow marks the parasite. Scale bar 200 μm.

Immunohistochemistry was performed on the last bird (AC2176) detected in 2025, revealing viral antigen in all examined tissues (Fig. 2). In the brain, neuronal clusters were positively immunolabelled, including Purkinje cells and cells of the molecular layer and the granular layer of the cerebellum. In the heart, viral antigen was detected within myocardial fibers, and in the lung, viral antigen was localized in endothelial cells of air capillaries. In the spleen, scattered positive cells were identified in the splenic pulp. Immunostaining was also evident in the kidney, where antigen was localized in the cytoplasm of tubular epithelial cells. Additionally, renal flukes were incidentally detected, with lymphocytic infiltrates surrounding the parasitized collecting duct. In the liver, hepatocytes exhibited positive immunoreactivity. In the stomach, immunostaining was observed in the mucosa (gastric glands), muscularis mucosae, and submucosa. While in the small intestine, viral antigen was detected in Brunner's glands within the duodenal submucosa.

### 3.4 USUV serological detection in birds

In 2024, 122 sera from 23 sentinel chickens were analyzed by cELISA, yielding five positive sera from two sentinel chickens (WN22CR145, WN24CR007), all originated from Girona province (l'Alt Empordà county). Sentinel WN22CR145 tested positive from October to December, while WN24CR007 tested positive from November to December and showed a doubtful result in September.

In 2025, surveillance was expanded in Girona province, and 104 sera from 10 sentinel chickens and 70 backyard poultry were analyzed by cELISA. Of these, 34 sera from sentinel chickens were collected from Castelló d'Empúries municipality. The same two positive sentinels from 2024 remained positive by cELISA in 2025. The sentinel WN24CR007 tested positive/doubtful from January to July 2025, and WN22CR145 continued positive from January to March 2025 when it died. The newly introduced sentinel chickens in September remained negative.

With regards to surveillance in backyard poultry five out of 30 *Anas* sp. from el Far d'Empordà municipality resulted positive by cELISA (two in May and three in July), and four were doubtful (July). In Ventalló municipality, 10 *Gallus gallus* broilers resulted all negative. In Fontanilles municipality, 30 *Anas* sp. were analyzed, resulting in one positive (August), and seven doubtful samples (one May, five in August and one in September).

All positive and doubtful sera were tested by SNT (Table 5). Sentinel bird WN24CR007 exhibited WNV-neutralizing antibodies in December 2024, however, from January 2025 onward, USUV-positive titres were higher than for WNV, which were negative, indicating USUV circulation. Sentinel WN22CR145 showed WNV and BAGV titres in November 2024, but specific viral infection could not be determined because one titre was not fourfold or higher than the other. In December 2024, WNV titre was also recorded, while USUV sample was cytotoxic and could not be evaluated. From January to March 2025, WN22CR145 displayed more than fourfold higher USUV-titres than WNV titres, indicating USUV circulation.

**Table 5:** Serum neutralization test results for two sentinel chickens positive by cELISA from Girona province in 2024 and 2025.

	Sentinel chicken WN24CR007			Sentinel chicken WN22CR145		
Date	WNV	BAGV	USUV	WNV	BAGV	USUV
24/09/2024	Negative	Negative	No data	No data	No data	No data
29/10/2024	No data	No data	No data	Negative	Negative	No data
25/11/2024	Negative	Negative	Negative	**Positive (1/40)**	**Positive (1/20)**	Negative
11/12/2024	**Positive (1/40)**	Negative	Negative	**Positive (1/40)**	Negative	Cytotoxic
31/01/2025	Negative	Negative	**Positive (1/80)**	**Positive (1/20)**	Negative	**Positive (1/160)**
24/02/2025	Negative	Negative	**Positive (1/40)**	**Positive (1/40)**	Negative	**Positive (1/160)**
25/03/2025	Negative	Negative	**Positive (1/10)**	**Positive (1/10)**	Negative	**Positive (1/160)**
29/04/2025	Negative	Negative	Negative	No data	No data	No data
28/05/2025	Negative	Negative	Negative	No data	No data	No data
26/06/2025	Negative	Negative	Negative	No data	No data	No data
23/07/2025	Negative	Negative	Negative	No data	No data	No data

WNV: West Nile virus, BAGV: Bagaza virus, USUV: Usutu virus.

All backyard bird sera tested SNT-negative for WNV, BAGV, and USUV, except for three *Anas* sp.; one from el Far d'Empordà and two from Fontanilles, that were weak USUV-positive. The respective blood samples were collected in July and August 2025, respectively.

Collectively, these results indicated flavivirus circulation and specifically USUV circulation in Girona province (Fig. 1) during the highest mosquito activity months.

### 3.5 USUV detection in mosquitoes

In 2025, 1344 mosquitoes from three species, 690 *Culex molestus*, 621 *Cx. pipiens* and 33 *Culex theileri* were tested in 84 mosquito pools. USUV was detected in one *Cx. pipiens* pool of 14 mosquito females from Castelló d'Empúries in late September (Fig. 1), the same area where USUV-positive SNT titres had been recorded in sentinel chickens.

### 3.6 Phylogenetic analysis of USUV

**Fig. 3:**
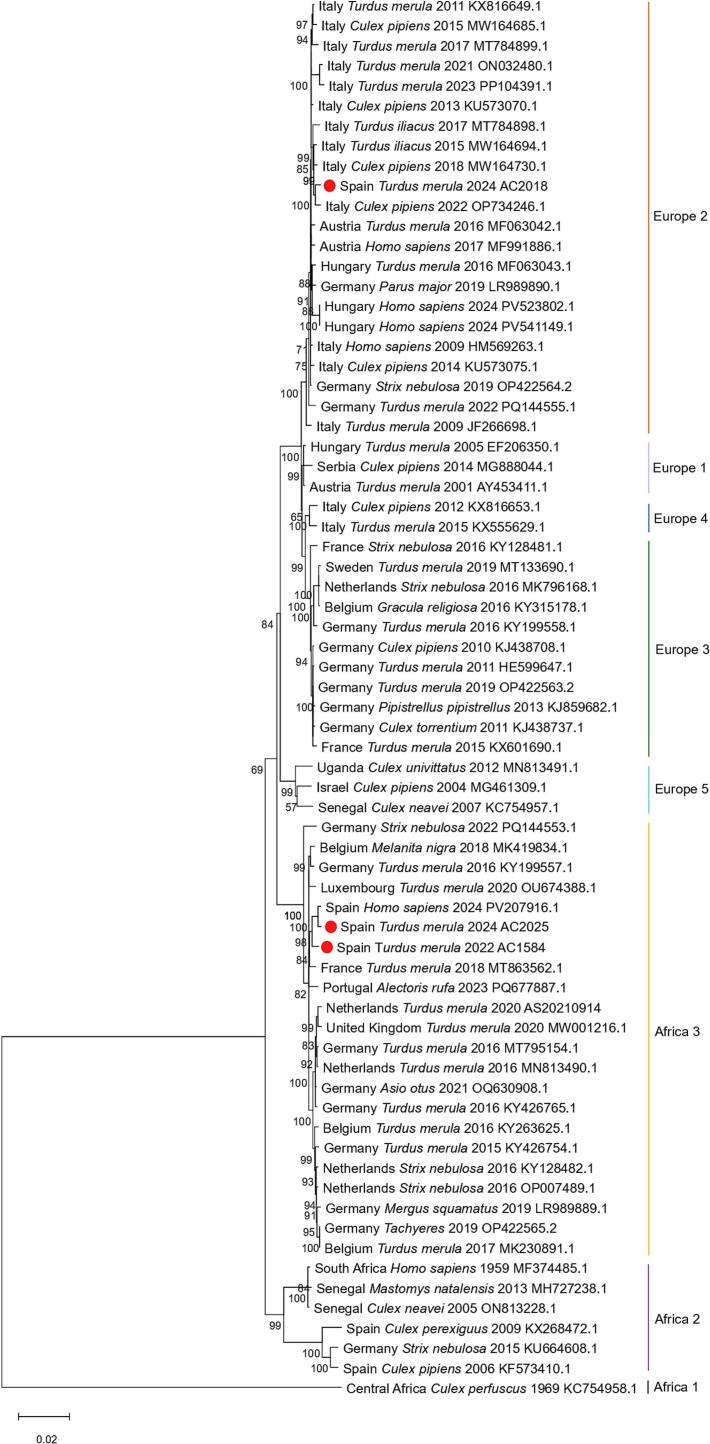
Phylogenetic tree of USUV virus generated using the Maximum Likelihood method and Tamura-Nei (1993) model [24] of nucleotide substitutions and the tree with the highest log likelihood (−36,267.03) was obtained. The percentage of replicate trees in which the associated taxa clustered together (1000 replicates) is shown next to the branches [40]. The initial tree for the heuristic search was selected by choosing the tree with the superior log-likelihood between a Neighbor-Joining (NJ) tree [41] and a Maximum Parsimony (MP) tree. The NJ tree was generated using a matrix of pairwise distances computed using the Tamura-Nei (1993) model [24]. The MP tree had the shortest length among 10 MP tree searches; each performed with a randomly generated starting tree. The analytical procedure encompassed 70 coding nucleotide sequences 1st, 2nd, 3rd, and non-coding positions with 10,305 positions in the final dataset. Evolutionary analyses were conducted in MEGA12 [25] utilizing up to 4 parallel computing threads.

A total of five out of six USUV-positive samples were successfully isolated and submitted for sequencing, yielding three complete genomes (one from 2022 and two from 2024) and one partial NS5 sequence (1655 nucleotides) from 2025. The phylogenetic analysis (Fig. 3) assigned, two isolates (AC1584 from 2022 and AC2025 from 2024) to the African 3 lineage, showing a close phylogenetic relationship with a human isolate detected in Barcelona in 2024 and clustering with French and Portuguese isolates. The third isolate (AC2018 from 2024) belonged to the Europe 2 lineage, marking its first reported detection in Spain, and clustered with Italian isolates (2015–2022).

The AC2149 and USUTU-33/25 (from positive mosquito pool) partial sequences showed 99% nucleotide identities to the Europe 2 isolate AC2018 from 2024, suggesting that they also belonged to Europe 2 lineage.

## 4 Discussion

This study demonstrates the co-circulation and overwintering of different USUV lineages in Northeastern Spain (2022–2025) through the integration of several components of surveillance within a One Health framework. USUV detection in wild, sentinel, and backyard birds, as well as in mosquitoes, confirms the previous existence of an active enzootic transmission cycle in a region where USUV has been detected in humans. This offers a more complete picture of USUV circulation.

Molecular detection of USUV in wild birds was restricted to blackbirds, despite targeted sampling of several bird species previously reported as USUV-positive in Europe. Although blackbirds represented only 4,85% of the dataset, six out of 18 individuals were positive, indicating a species-specific susceptibility or amplification role which aligns with existing literature [26], [27]. These findings expand previous serological evidence of USUV in Northeastern Spain [14] by providing the first molecular confirmation of USUV in wild birds.

The detection of USUV RNA in multiple organs in a naturally infected blackbird (AC2176) provided evidence of systemic infection. This was further supported by the widespread distribution of viral antigen in multiple organs observed by immunohistochemistry, This pattern closely mirrors findings from a recent experimental infection in blackbirds [28], where USUV showed broad tissue tropism and systemic infection, including marked involvement of visceral organs and the central nervous system. The high viral loads detected in feathers in AC2176 aligned with studies on naturally infected birds identifying irrigated feathers as valuable sample for diagnosis [29], [30]. While molecular detection of USUV in wild birds was confirmed only in Barcelona province, serological and virological surveillance in poultry and mosquitoes demonstrated USUV circulation in Girona province. These findings indicate a broader USUV distribution than that detected by wild-bird surveillance alone and highlight the importance of integrating several surveillance components to detect USUV circulation. The absence of molecular detection in wild birds from other provinces, despite prior serological evidence [14], is likely related to limited sample sizes, transient or low viremia in birds, and species-specific susceptibility. The lack of detections in 2021 and 2023 may reflect low viral circulation and interannual variability, potentially associated with fluctuations in vector activity, as suggested in previous USUV studies [4]. However, sampling limitations cannot be ruled out.

Temporally, USUV detection in wild birds and mosquitoes occurred in late summer and early autumn, consistent with the vector activity period in Catalonia [15] and reports from other European countries [12]. Serological findings in sentinel and backyard birds during 2024–2025 provided information on the timing of USUV exposure. The initial detection of antibodies by cELISA, a highly sensitive screening assay, likely reflects the first exposure of sentinels in September and backyard birds in late July–August. The persistence of USUV neutralizing antibodies in sentinel birds throughout winter and spring most plausibly represents residual immunity, especially in the absence of new seroconversions in newly introduced sentinel birds. Cross-reactive neutralization with WNV and BAGV underscored the complexity of flavivirus serology [14]. In this context, the co-circulation of flaviviruses, including USUV and WNV, has important implications for diagnosis and surveillance, as these viruses belong to the same serocomplex and may exhibit cross-reactivity in serological assays used in both human and animal screening [31]. In areas where multiple flavivirus co-circulate, serological findings may not reliably distinguish between infections, potentially leading to misclassification or inconclusive results [14], [31]. These highlight the importance of confirmatory neutralization assays and the integrated interpretation of serological and molecular data.

Phylogenetic analysis revealed the co-circulation and overwintering of two USUV lineages in Catalonia; Africa 3 (detected in 2022 and 2024) and Europe 2 (detected in 2024 and 2025), reflecting the possibility of the virus persisting in diapausing mosquitoes or circulating in resident bird populations during winter in Catalonia as previously suggested for other regions [10]. Moreover, we did not detect Africa 2 detected 20 years ago [11], suggesting the extinction of this lineage. Two of the USUV isolates (AC1584 and AC2025) belonged to the Africa 3 lineage, a genotype already reported in Europe since 2014 [12], [32] and in Spain in 2018 [33]. These isolates clustered closely with a human USUV strain from Barcelona in 2024 [34], indicating that USUV overwintered and circulated among wild birds before 2024. This pattern supports a human spillover rather than a new USUV introduction in 2024. In contrast, isolates AC2018 and AC2149 were assigned in the Europe 2 lineage, first detected in Italy in 2009 [35] and later spread across several Central and Southern European countries [21]. Its identification in Catalonia constitutes the first detection in Spain, highlighting its westward expansion in Europe.

The two blackbirds that could be evaluated alive, both infected with the Africa 3 lineage, exhibited poor body condition and neurological signs, along with pathological findings (including splenomegaly, pale kidney, pulmonary haemorrhage and lung lesion) consistent with USUV disease as described in natural [29] and experimental infections [28]. These observations provide further evidence that the Africa 3 lineage can cause clinical disease and even death in blackbirds. An emaciated blackbird infected with a Europe 2 strain was found dead without evident gross lesions. Similar findings have been described in Europe 2-infected blackbirds, which often show poor body condition but without consistent macroscopic pathology [36]. In contrast, marked pathological alterations linked to this lineage have been documented in blackbirds in the literature [37]. These included splenomegaly, hepatomegaly and visceral congestion, together with histopathological evidence of hepatic necrosis and splenic lesion. In line with the latter observations, the Europe 2-infected blackbird in 2025 showed a severe multiorgan clinical presentation. These findings indicate that Europe 2 infection can result in variable pathological outcomes, from minimal lesions to multisystemic disease. Overall, these results suggest potential differences in pathogenesis between USUV lineages; however, the limited number of cases does not allow definitive conclusions.

USUV is currently widespread across Europe, increasing human infection risk. Although no transfusion-transmitted cases have been confirmed, repeated viral detection in blood donors raises blood-safety concerns [38]. USUV's similarity to WNV suggests a potential risk for transmission through blood and organ donations [38]. Although usually found in asymptomatic blood donors, the Africa 3 and Europe 2 lineages circulating in Catalonia have caused human infections, including severe cases in immunocompromised individuals [5], [13], [39]. Ongoing surveillance and mosquito control are needed, especially given the confirmed human spillover and evidence of the virus overwintering.

## 5 Conclusion

Circulation of USUV in Northeastern Spain was confirmed between 2022 and 2025. USUV infection was detected in resident blackbirds, supporting their role as a highly susceptible host and key amplifiers. Phylogenetic analyses revealed the co-circulation and overwintering of two distinct lineages (Africa 3 and Europe 2), with Europe 2 marking its westward expansion in Europe. The presence of lineages previously associated with human infections, together with evidence of overwintering and confirmed human spillover, highlights the epidemiological and public health relevance of USUV in Spain. Overall, these findings demonstrate the value of integrated surveillance and support the implementation of a One Health approach, combining wildlife, poultry, vector and human health monitoring apart from vector control to mitigate future USUV emergence and spread.

## CRediT authorship contribution statement

**Arjola Leka:** Writing – original draft, Investigation, Formal analysis. **Jaume Gardela:** Writing – review & editing, Investigation, Formal analysis. **Elena Obón:** Writing – review & editing, Methodology, Investigation. **Maria Pifarré:** Writing – review & editing, Methodology, Investigation. **Miguel Julián Martínez:** Writing – review & editing, Validation. **Jéssica Navero-Castillejos:** Writing – review & editing, Validation. **Ramon Casals:** Writing – review & editing, Resources, Methodology. **Alba Solé:** Writing – review & editing, Resources. **Núria Busquets:** Writing – review & editing, Writing – original draft, Supervision, Resources, Project administration, Methodology, Investigation, Funding acquisition, Formal analysis, Data curation, Conceptualization.

## Funding details

This work was supported by Centres de Recerca de Catalunya (CERCA), the 10.13039/501100010552Departament de Salut, Generalitat de Catalunya; and the 10.13039/501100002809Departament d'Agricultura, Ramaderia, Pesca i Alimentació, Generalitat de Catalunya.

## Declaration of competing interest

The authors declare that they have no known competing financial interests or personal relationships that could have appeared to influence the work reported in this paper.

## Data Availability

Data will be made available on request.

## Appendix A Supplementary data

**Figure S1.** Distribution of bird samples A) by province B) across orders and species.

**Video S1:** Blackbird AC2025 exhibited repetitive head movements during clinical examination.
