# Supplementary figures and images for: Circulation and overwintering of Usutu virus lineages in north-eastern Spain: A one health perspective (2021–2025)
Source: One Health. 2026 Apr 1;22:101400. doi: 10.1016/j.onehlt.2026.101400 (PMC13089210; doi:10.1016/j.onehlt.2026.101400)

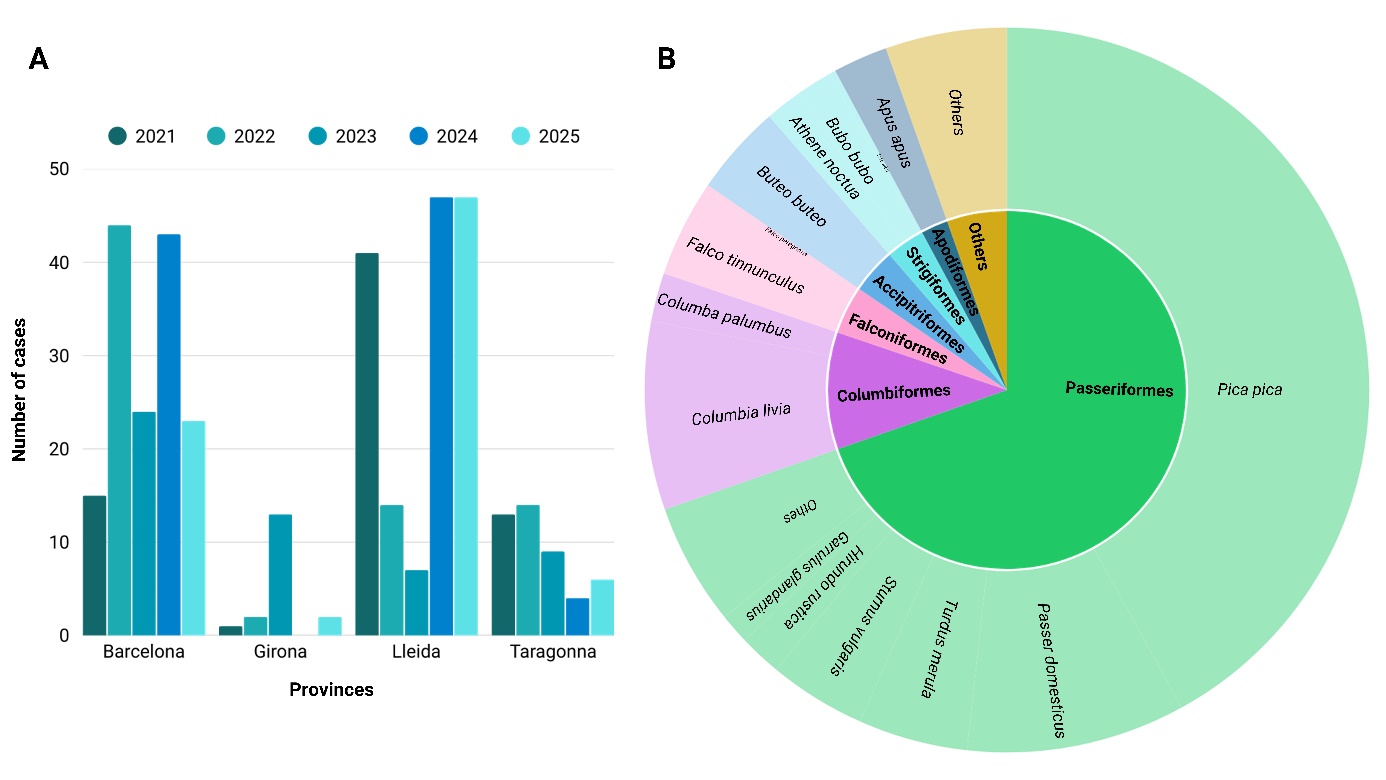


**Figure S1.** Distribution of bird samples A) by province B) across orders and species.

Supplement: Supplementary file 1 — Figure S1. Distribution of bird samples A) by province B) across orders and species. [file mmc1.docx]
